# Avulsion of Permanent Mandibular Incisors: A Report of Two Cases with Pertinent Literature

**DOI:** 10.1155/2023/6204171

**Published:** 2023-05-04

**Authors:** Ibadat Preet Kaur, Jitendra Sharan, Pallawi Sinha, Ashok Kumar, Anand Marya

**Affiliations:** ^1^Department of Dentistry, ESI Medical College and Hospital, Alwar, Rajasthan, India; ^2^Unit of Orthodontics and Dentofacial Orthopedics, Department of Dentistry, All India Institute of Medical Sciences, Bhubaneswar, Odisha, India; ^3^Department of Prosthodontics, Hi-Tech Dental College and Hospital, Bhubaneswar, Odisha, India; ^4^Department of Pedodontics and Preventive Dentistry, ESIC Dental College and Hospital, Rohini, New Delhi, India; ^5^Department of Orthodontics and Dentofacial Orthopedics, Faculty of Dentistry, University of Puthisastra, Phnom Penh, Cambodia; ^6^Center for Transdisciplinary Research, Saveetha Dental College, Saveetha Institute of Medical and Technical Science, Saveetha University, Chennai 600077, India

## Abstract

*Introduction.* This study reports two rare cases of avulsion of permanent mandibular incisors with their sequelae after being reimplanted by two contrary methods. The relevant literature regarding the avulsion of permanent mandibular incisors is also being discussed. *Case Presentation.* In Case I, a 9-year-old girl reported an avulsion of the permanent mandibular left lateral incisor that was immediately reimplanted within 20 minutes after injury, whereas in Case II, all four permanent mandibular incisors were avulsed and reimplanted after a prolonged extraoral dry time of 36 hours in an 18-year-old female. Both cases missed their scheduled follow-up visits and were reported after 3.5 years and 7 months, respectively, with severe root and alveolar bone resorption that was confirmed by clinical examination and Intra oral periapical radiograph (IOPA) radiographs. *Discussion.* Avulsion of permanent mandibular incisors is rare. The similar unfavorable outcome of contrary cases at a variable duration of time after missed follow-up illustrates the role of the appropriate treatment protocol and regular follow-up visits for the long-term success of reimplanted teeth.

## 1. Introduction

Dental traumatic injury is a sudden, circumstantial, unexpected, accidental impact injury to teeth and/or other hard and soft tissues within and around the vicinity of the oral cavity that often requires emergency attention [1]. Tooth avulsion is the complete displacement of a tooth from its socket and has a prevalence of 0.5–3% of traumatic injuries in permanent dentition [2]. Avulsion is more common in men than in women, and its prevalence increases between the ages of 7 and 9 years due to loose PDL around incompletely developed roots, which provides minimal resistance against extrusive forces during the eruption period of teeth [1, 3]. It has a maximum predilection for the maxillary central incisors, followed by maxillary laterals and mandibular central and lateral incisors [4, 5]. Multiple avulsions of the teeth are mostly reported with concomitant hard and soft tissue injuries in severe accidents and assaults [4–6].

The avulsion of permanent mandibular incisors is quite rare, with only 15 cases reported in literature till 1st December 2022 (Table 1). Two categories of cases have been recognized according to their nature, i.e., direct: the one primarily reporting the reimplantation of avulsed permanent mandibular incisors (Case Nos. 1–10) and indirect: the cases primarily reporting the consequences and rehabilitation after the avulsive loss of permanent mandibular incisors (Case Nos. 11–15). Amongst them, seven cases (Case Nos. 1–4 and 7–9) have recorded successful outcomes after follow-up for a minimum of one year after reimplantation.

The present study adds to the literature two rare cases of avulsion of permanent mandibular incisors according to the CARE guidelines [21]. Case I describes single mandibular lateral incisor avulsion, and Case II, is the first case to date reporting the avulsion of all four mature permanent mandibular incisors in an adolescent female.

## 2. Case Reports

### 2.1. Case I

**Figure 1:**
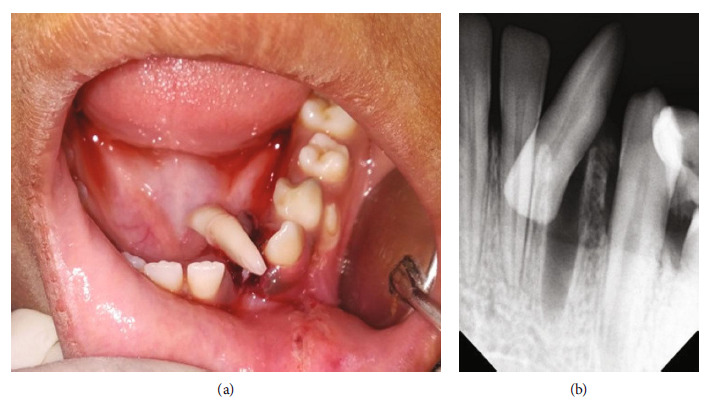
Case I: preoperative photographs of 9-year-old female presenting with avulsed tooth #32 attached to gingiva in the oral cavity. (a) Intraoral. (b) IOPA.

**Figure 2:**
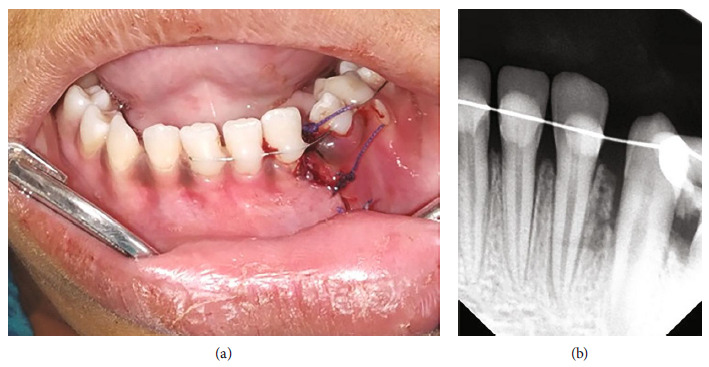
Case I: immediate post-operative photographs of reimplanted and stabilized tooth #32. (a) Intraoral. (b) IOPA.

**Figure 3:**
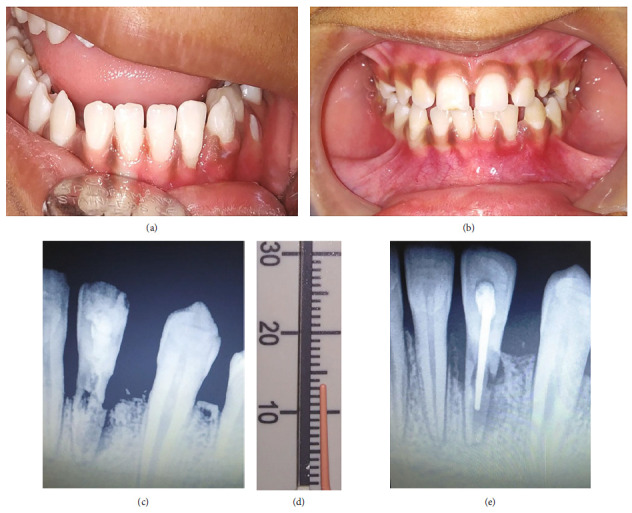
Case I: follow-up. (a) Frontal view after removal of splint at 2 weeks with adequate healing. (b) Frontal view showing slight inflammatory redness around tooth #32 after 3.5 years. (c) Radiograph showing root resorption till middle one-third of root at 3.5 years. (d) No. 80 Gutta Percha adjusted to 13 mm according to the suspected length of periodontal communication. (e) 20° angulated radiograph showing severe communicating internal and external root-resorption at 3.5 years.

**Figure 4:**
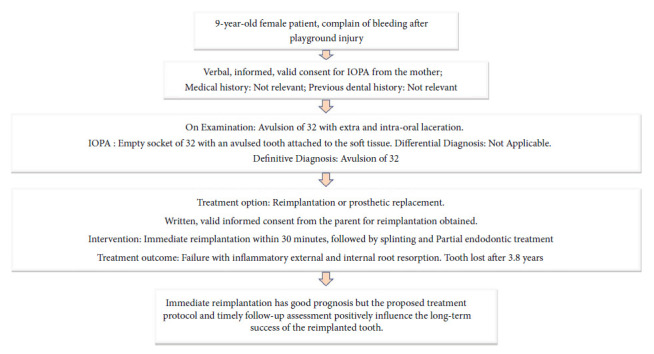
Procedure checklist and their chronological order of treatment and follow-up of Case I.

A 9-year-old healthy female patient reported to the dental department with chief complaint of bleeding from face and oral cavity due to a direct impact with a ball in the playground. The incident occurred 10 minutes earlier, resulting in an extraoral and intraoral laceration and an avulsion of the permanent left mandibular lateral incisor (i.e., tooth #32) without any associated hard tissue injury. An avulsed tooth was attached to the gingival tissue in the oral cavity (Figure 1(a)). An intraoral radiographic examination ruled out presence of an associated alveolar fracture (Figure 1(b)). To save crucial minutes, the tooth was immediately placed back in the socket within 20 minutes of injury. The tooth reimplantation and alternative replacement options were explained in detail to her mother, and written informed consent for the reimplantation was obtained from her. The permanent mandibular lateral incisor was accurately repositioned with slight digital pressure. It was stabilized from teeth #41 to #75 with 28-G stainless steel wire, and acid etch composite resin. The lacerations (Figures 2(a) and 2(b)) were also sutured using non-absorbable sutures. The combination of 250 mg of amoxicillin and 125 mg of clavulanic acid twice a day along with 0.1% chlorhexidine mouth rinses thrice a day, were prescribed for five and seven days, respectively. The parent was repeatedly informed about the severity of the condition and the significance of subsequent follow-up visits for further treatment. She was finally referred for consideration regarding a tetanus booster. At the first weekly follow-up, sutures were removed, and intraoral access opening was prepared without local anesthesia. Keeping the working length of 18 mm recorded electronically, a thorough debridement was done until the No. 60 K file. After copious irrigation with 3% sodium hypochlorite and normal saline, a thick paste of calcium hydroxide was placed in the canal. The access cavity was sealed with high-density glass ionomer cement. An intraoral splint was removed after 2 weeks (Figure 3(a)). The tooth in question was stable and completely asymptomatic, and hence next follow-up for endodontic management was scheduled after 2 weeks. However, the patient missed her appointment and reported after 3.5 years with the complaint of pain in the reimplanted tooth. Clinical examination recorded a tender, grade I mobile tooth with visible gingival inflammation, and deep periodontal pockets (Figure 3(b)). The Intra oral periapical radiograph (IOPA) of the tooth recorded severe inflammatory root resorption until the middle one-third of the root (Figure 3(c)). The root canal was re-assessed, and a 5 mm loss of working length was recorded electronically, suggesting the presence of periodontal communication. A 20° horizontally angulated IOPA with a 13 mm gutta-percha point confirmed the presence of severe inflammatory external and internal resorption in the reimplanted tooth (Figures 3(d) and 3(e)). The parents refused the extraction of the tooth. It exfoliated on its own during function after 3 months. The timeline, chronological order of treatment, and follow-ups are described in Table 2 and Figure 4.

### 2.2. Case II

**Figure 5:**
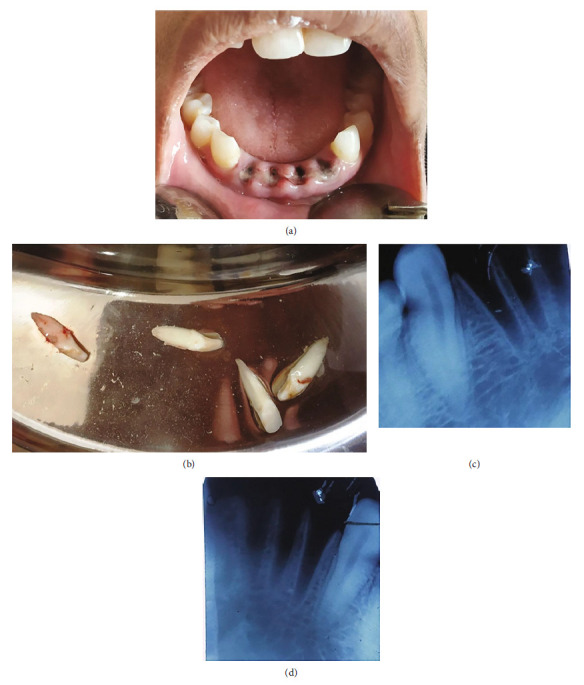
Case II: pre-operative photographs of an 18-year-old woman with avulsion of teeth #31, #32, #41, and #42. (a) Intraoral. (b) Avulsed teeth. (c) IOPA of the teeth #31 and #32. (d) IOPA of the teeth #41 and #42.

**Figure 6:**
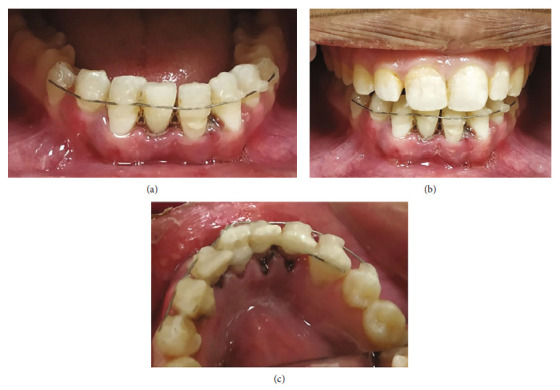
Case II: immediate post-operative photographs of reimplanted and stabilized teeth #31, #32, #41, and #42. (a) Frontal view of mandibular anterior teeth. (b) Frontal view with maximum intercuspation. (c) Lingual view of mandibular anterior teeth.

**Figure 7:**
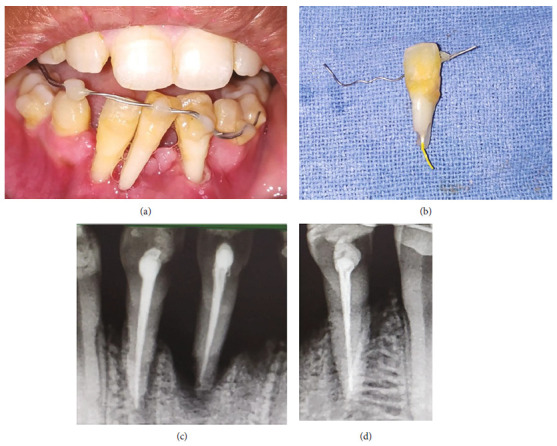
Case II: follow-up at 7 months. (a) Frontal view of mandibular anterior teeth showing fractured splint and periodontally compromised teeth #32, #41, and #42. (b) Re-avulsed tooth #31 with root resorption till the middle third of root and calculus deposits below cemeto-enamel junction. (c) IOPA showing inflammatory root and bone resorption of teeth #41 and #42. (d) IOPA showing inflammatory root and bone resorption with reference to tooth #32.

An 18-year-old female patient reported a complaint of loss of four mandibular incisors, secondary to an elliptic seizure and fall, which happened 36 hours back. Following the fall, primary care was provided at a local hospital, and she was referred to the higher center for a final opinion regarding the avulsed teeth. Clinical evaluation has revealed that the teeth were completely dry, sockets were filled with coagulum (Figures 5(a) and 5(b)), and absence of any associated alveolar bone fracture (Figures 5(c) and 5(d)). Possible treatment options, risks, and prognosis were explained in detail, and written informed consent for delayed reimplantation was obtained. After removing necrotic and dried remnants of the periodontal ligament with a sterile gauge, RCT was carried out extraorally. Under local infiltration with 2% lignocaine, the coagulum of the sockets was gently curetted and rinsed with saline solution. The avulsed teeth were gently placed with digital pressure in their respective sockets and stabilized with a labial splint from teeth #34 to #44 using 32-G twisted orthodontic ligature wire and composite resin. They were also reinforced with an additional lingual splint from teeth #33 to #43 using 30-G SS wire (Figures 6(a), 6(b), and 6(c)). Post-stabilization antibiotics and mouthwash were prescribed. She was advised to be on a soft diet and maintain optimal oral hygiene. The weekly follow-up was scheduled, and medical consultation regarding the sudden onset of seizures. However, she missed her regular follow-up visits and reported after 7 months with a fractured splint (Figure 7(a)).

**Figure 8:**
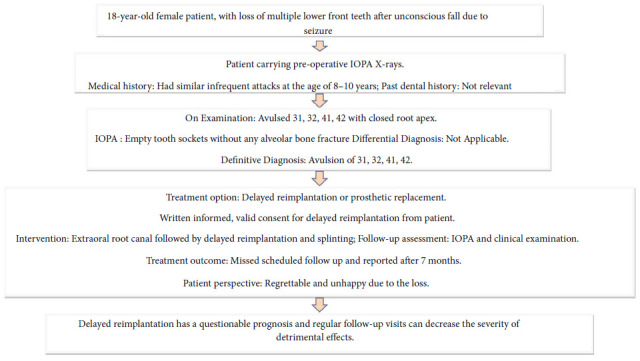
Procedure checklist and their chronological order of treatment and follow-up of Case II.

Clinically, tooth #31 was re-avulsed with severe root resorption (Figure 7(b)). Deep periodontal pockets and grade III mobility were recorded for the remaining implanted teeth. The inflammatory root-resorption with severe alveolar bone loss was also evident on the IOPA's of remaining teeth, suggesting the procedure's failure (Figure 7(c)). She was referred for extraction of teeth #32, #41, and #42, followed by oral rehabilitation. The timeline, chronological order of treatment, and follow-up are described in Table 3 and Figure 8.

## 3. Discussion

Avulsion is one of the most serious forms of traumatic dental injury demanding reimplantation for the survival of the exarticulated tooth [1, 22]. Extraoral storage time and storage medium are the most critical factors for the survival of PDL cells that determine the long-term prognosis of the reimplanted tooth [22, 23]. Both are the foundation for the three defined categories of International Association of Dental Traumatology (IADT) guidelines, i.e., PDL cells-most likely viable, compromised but viable, and non-viable, in descending order of expected long-term survival of the avulsed and reimplanted tooth [22, 23].

Playground trauma, an etiological factor for Case I, is identified as one of the common causes of dental avulsion in children. Though epileptic patients are more prone to oral and maxillofacial trauma than healthy individuals, dental avulsion is uncommon and frequently documented as the avulsion of multiple maxillary teeth in them [24, 25]. The literature review also confirms that Case II is the first case to date reporting the avulsion of all four mature mandibular incisors in an adolescent female after a fall due to an infrequent etiological factor, i.e., an epileptic seizure. Both cases were classified and attempted to be treated according to the revised 2012 IADT guidelines for managing avulsed teeth, considered the best evidence for managing traumatic dental injuries [23].

In Case I, the PDL cells were most likely viable as the patient reported immediately after trauma with a tooth stored by chance in her saliva. Saliva is an established physiological storage medium, preferably for 30 minutes after avulsion, as its composition significantly damages the functional capacity of PDL cells after one hour [26]. Composite splints with a diameter of 0.3–0.4 mm are considered flexible splints that decrease the rate of ankylosis by allowing functional physiological movement [27]. Therefore, a 28 G (0.32 mm diameter) acid etch composite wire splint was used for 2 weeks due to its availability in the department. The tooth was completely stable after the removal of the splint suggesting adequate periodontal healing at that time.

Due to a prolonged extraoral dry time of approximately 36 hours, Case II was treated as delayed reimplantation [23]. Although the probability of long-term survival was low due to negligible surviving PDL cells, a joint decision of reimplantation was made considering the irreversible consequences of loss of four permanent mandibular incisors in the young female patient. Endodontic treatment was completed extraorally, and teeth were reimplanted and stabilized with a flexible labial splint [27]. As they were graded as unstable after the labial splint, an additional splint was placed on the lingual side. IADT recommends semi-rigid splinting for 4 weeks for delayed reimplantation, leaving the final decision upon the clinician [22, 23]. The literature does not mention the evidence for the splinting procedure, time, and duration of multiple avulsed teeth. Hence, the best appropriate clinical decision was taken according to the situation. The splints were intended to be removed according to the adequate organization of periodontal tissues.

Both patients missed their scheduled follow-up visits and reported after different periods with severe resorption and mobility in reimplanted teeth suggesting the failure of reimplantation procedures. The outcome of reimplanted teeth is quite unpredictable, with varied success rates ranging from 4% to 50% in different studies [28–31]. The current evidence reports a significantly greater relative risk of failure in delayed reimplantation with prolonged extraoral dry time, delayed pulp extirpation (after 20 days), immature teeth, patients younger than 11 years, and teeth requiring prolonged calcium hydroxide therapy [31]. Common complications that may occur weeks, months, or even years after avulsion are pulp necrosis, root resorption, and ankylosis [32]. The reported incidence of root resorption related to avulsed teeth is highest for replacement resorption and ankylosis (51%), followed by inflammatory (23.2%), and internal root resorption (1.2%) [33]. The underlying mechanisms for such resorptions are not completely understood; evidently, it is found that the mechanisms in inflamed pulp tissue initiate inflammatory and internal resorption, whereas the injury and hypoxia of PDL cells institute external root resorption in traumatic teeth [34].

Case I declined further treatment after the placement of calcium hydroxide and reported after 3 years of internal and external inflammatory root resorption in an endodontically initiated reimplanted tooth. Considering the etiopathogenesis, the appearance of internal resorption after complete pulpectomy is an exceptional presentation [33, 34]. For a tooth with closed apex considered for immediate reimplantation, IADT recommends beginning endodontic treatment within 7–10 days of reimplantation [22, 23]. The prepared canal is treated with calcium hydroxide for up to one month, followed by its filling with an acceptable material [23]. Incomplete endodontic treatment without final sealing of the root canal undoubtedly culminated present clinical situation [31, 35]. The antimicrobial activity of calcium hydroxide is directly proportional to the maintenance of its high pH value. The presence of inflammatory exudates, necrotic pulp tissue remnants, and the buffering effect of dentinal hydroxyapatite eventually decreased it [36]. The diminished efficacy of intracanal medicament and the cemental damage on the external root surface exaggerated the ongoing inflammatory processes from within and outside the tooth. This vicious cycle of silent internal and external inflammatory root resorption resulted in a severely resorbed asymptomatic reimplanted tooth for 3.5 years. The anticipated long-term prognosis of Case II was already poor following the current evidence, and the patient missed her follow-ups for assessment and splint removal. In contrast to the increased incidences of replacement resorption and ankylosis reported for longer splinting periods of the tooth [35, 37], massive inflammatory root resorption within 7 months after reimplantation was present in the second case. It is attributed to the multiple avulsed teeth extensive PDL and cemental damage. Established calculus deposits on the resorbed teeth indicate that the prolonged composite splints compromised the ability to maintain oral hygiene and served as the nidus for plaque accumulation. The bacterial toxins stimulated the clastic cells and further amplified the rapidly progressing inflammatory processes resulting in the rapid resorption of reimplanted teeth within a short duration [33, 34].

The present case highlights the significance of adequate follow-up visits and appropriate patient compliance for the long-term success of reimplanted avulsed teeth. Given immediate reimplantation following recommended guidelines, which resulted in a completely asymptomatic tooth at the time of splint removal, a favorable long-term outcome was expected in Case I. The tooth remained functional for approximately 3.7 years, and the duration had certainly been prolonged if the initial stability had not been assumed as the final result and endodontic treatment would have been completed in time. On the contrary, the severe injury to multiple teeth with exceptionally prolonged extraoral dry time expectedly worsened the prognosis from the start of the second case. Though unintentionally, follow-up visits had been missed, and reimplanted teeth were lost within 7 months due to rapid inflammatory resorption of root and alveolar bone. Müller et al. reported a mean survival duration of 1.7 years for reimplanted teeth affected by inflammatory resorption as compared with 6.1 years by replacement resorption. It has also been concluded that they are likely to remain functional if no signs of resorption are evident within 3 years of reimplantation, but are intended to be lost even after the years of treatment if resorptive changes appear within 3 years [38]. Considering the missed follow-up as the limiting factor, both cases were expected to be lost in the long term as the signs of resorption are evident within 3 years. As inflammatory root resorption can be radiographically diagnosed in the shortest duration of 1 month [29], the consequences would have been less catastrophic in both cases if adequate follow-up visits had been maintained and the teeth would have been extracted only at the initial signs of failure and before the establishment of severe bone resorption. Another limiting factor for Case II is reimplanting multiple teeth after an excessively prolonged extraoral dry time without additional efforts to arrest root resorption. The specific upper limit of extraoral dry time for delayed reimplantation has not been defined. Several modalities, including surface treatments, retrograde fillings, and even the obturation of a complete root canal with bioactive materials to arrest root resorption of delayed reimplanted teeth, have been documented with acceptable results in the literature [39, 40]. Due to the limited data and insufficient clinical trials, there are no strict recommendations about these in IADT 2012 [23] and even recently modified 2020 guidelines [22, 41]. This warrants considerable research in this field, so that cases with poor prognoses can be managed with more acceptable outcomes in the future.

## 4. Conclusions

The avulsion of permanent mandibular incisors is rare, with unpredictable outcomes. IADT recommended protocol for reimplantation with timely follow-up visits positively influences the success and decreases the detrimental effects in case of failed reimplanted teeth.

## Figures and Tables

**Table 1:** Case reporting avulsion of the permanent mandibular incisors.

Case no.	Nature	Mandibular tooth avulsed		Age (years)	Etiology	Extraoral time	Storage media	Treatment	Follow-up	Outcome (at final follow-up)	Author/year
		S. no.	FDI notation								
1	Direct	1	#31	11	Playground injury	3 hours	Dry	Delayed implantation (DR) after soaking in doxycycline	2 years	Success—arrested root resorption	Calişkan et al. [7]
2	Direct	2	#31	6	Fall	2 hours	Milk	DR	8 years 8 months	Success—surface resorption in both	Kinoshita et al. [8]
3	Direct	3	#41	9	Fall	11 hours	Dry	DR	1 year 6 months		
4	Direct	4	#42	12	Playground injury	20 minutes	None	Self immediate implantation (IR). Late root canal treatment (RCT) after 6 months.	1 year	Success—asymptomatic and stable	Emerich et al. [9]
5	Direct	5	#41	12	Dog bite	5 hours	Entrapped in intraoral laceration	IR RCT after 1 week	1 week	Success—asymptomatic and stable	Bianco et al. [10]
6	Direct	6	#41	8	Baseball injury	6 hours	Milk	IR. No RCT	3 weeks	Failure—extraction and rehabilitation	Boynton and Barber [11]
7	Direct	7	#31	13	Self injury by thread	11 days	Dry wrapped in cloth	DR	20 months	Functional with replacement resorption	Shweta et al. [12]
8	Direct	8	#31	7	Fall	20 minutes	Milk	IR No RCT. Single-step regenerative therapy after 6 months for a failed reimplantation	2 years	Success—asymptomatic and stable continuous root development	Chaniotis [13]
9	Direct	9	#41	8	Playground injury	3 hours	Not stored	DR	4 years	Success—asymptomatic and stable	Abuhaimed [14]
		10	#42					RCT after 4 months			
10	Direct	11	#42	17	Domestic	Less than 30 minutes	Milk	IR after topical doxycycline application	6 months	Success—asymptomatic and vital on electric pulp testing testing	Sankar et al. [15]
					Accident						
11	Indirect	12	#31	11	Playground injury	15 days	Not stored	Not implanted (NR)	Not applicable (NA)	NA	Bonanato et al. [16]
								Replacement by fibre reinforced adhesive FPD			
12	Indirect	13	#31	12	Fall	No tooth found	NA	NR	NA	NA	Elbay et al. [17]
		14	#41					Prosthetic			
								Rehabilitation			
13	Indirect	15	#31	8	Playground injury	3 hours	Teeth not recovered	Fixed space maintainer	NA	NA	Salako et al. [18]
		16	#32								
		17	#41								
		18	#42								
14	Indirect	19	#31	13	Sledge	NR	NR	Reimplantation	NA	NA	Schneider and Moser [19]
		20	#32		Accident	NR	NR	No RCT	NA	NA	
								Extracted after 15 months. Orthodontic management of extraction spaces			
15	Indirect	21	#31	6	Fall	NR	NA	NR	4 year	Root-like structures found on incidental ortho pantomogram (OPG) examination	Reis et al. [20]
		22	#31					Prosthetic rehabilitation by removable partial denture			
		23	#41								
		24	#42								

**Table 2:** Timeline of Case I.

Time	Event	Symptoms
0	Patient-reported 10 minutes after injury in the playground with a complaint of bleeding from the oral cavity and face	An extraoral and intraoral laceration with an avulsion of tooth #32 was present
0	Immediate reimplantation with splinting and extraoral and intraoral suturing	
+1 week (first follow-up, physically present)	Access opening and bio-mechanical preparation followed by calcium hydroxide dressing done	Tenderness in the reimplanted tooth
	Sutures removed	
+2 weeks (second follow-up, physically present)	Splint removed	No pain or tenderness (symptom-free)
+4 weeks (third follow-up, telephonically)	Refused further treatment	No pain, swelling (symptom-free)
+3.5 years (fourth follow-up, physically present)	The patient visited with pain on biting food for 15 days. Tenderness on the percussion with grade I mobility and periodontal pockets. IOPA shows severe root and alveolar bone resorption. The apex locator records the loss of working length by 5 mm	Pain with inflammatory external and internal resorption on angulated IOPA. Failure of the reimplantation procedure
+3.8 years (fifth follow-up, telephonically)	Tooth lost during eating	

**Table 3:** Timeline of Case II.

Time	Event	Associated findings
0	The patient reported 36 hours after the loss of multiple front lower teeth after an unconscious fall due to a seizure	Moderate pain in anterior mandibular region present
	Empty sockets of teeth #31, #32, #41, and #42 were clinically and radiographically suggesting tooth avulsion	
0	Extraoral RCT and delayed reimplantation of four teeth were done. The teeth were stabilized with simultaneous labial and lingual splints	
	Scheduled weekly follow-up missed	
+7 months (first follow-up, physically present)	Patient reported a fractured splint, re-avulsed tooth #31, grade III mobile teeth #32, #41, and #42, and deep periodontal pockets	Severe inflammatory root and alveolar bone resorption suggest a failure of the reimplantation procedure

## Data Availability

Data supporting this research article are available from the corresponding author or first author on reasonable request.
